# Application of Digital Image Correlation (DIC) Method for Road Material Testing

**DOI:** 10.3390/ma12152349

**Published:** 2019-07-24

**Authors:** Jarosław Górszczyk, Konrad Malicki, Teresa Zych

**Affiliations:** Faculty of Civil Engineering, Cracow University of Technology, 31-155 Cracow, Poland

**Keywords:** digital image correlation, DIC technique, image analysis, tension test, displacement measurement, geogrid, geosynthetics

## Abstract

The theoretical part of the paper presents the framework of the digital image correlation (DIC) method as well as its advantages and limitations. The DIC technique that can be used in static and fatigue tests is a non-contact, non-interferometric optical method for measuring the surface deformation of structural elements, and material samples. In the experimental part of the paper, the implementation of the DIC method for the selected laboratory tests of building materials is described. The results of the tests on the samples of the materials used in road construction, i.e., asphalt mixtures (HMA), stone, soil stabilized with a hydraulic binder, and geosynthetics are discussed. The conducted research pointed out the possibilities of using the DIC method to evaluate the deformation of road materials in laboratory tests, taking into account their specificity. The variety of samples of tested material allowed to indicate the areas in which the DIC method and the algorithms used to evaluate the results give a significant advantage compared to tensometric measurement methods.

## 1. Introduction

The measurement of displacement/deformation at any point of a structural element/material subjected to external loads is important in many engineering applications. The obtained strain values are used to visualize the strength problems in a structural member.

The conventional instruments that measure strains (i.e., strain gauges, extensometers) cannot generate strain maps. A full-field strain measurement is possible using the Digital Image Correlation (DIC) technique. This method belongs to the group of optical, non-interferometric techniques, which determine deformation by comparing the changes in the image of the surface of a tested object before and after deformation [1]. The DIC technique enables new and more complex investigations.

The DIC technique was introduced in the 1980s [2,3,4,5]. Since then the quality of digital images has improved enormously and multiple major modifications of algorithms have been implemented. Nowadays the DIC method with high sensitivity and accuracy is utilized for testing specimens and, most recently, the whole structures. It can be used in laboratory or outdoor environments. The DIC techniques are applied not only in the field of engineering, but also in medicine, mining, conservation of cultural heritage, etc., [6,7,8,9,10,11].

The examples of the application of the DIC method for testing road materials are presented in the paper. At the beginning, the theoretical fundamentals, advantages, and limitations of the DIC method are described. Then the implementation of this method for laboratory testing of selected materials used in the road construction is presented. The results of the tests carried out on asphalt mixtures (HMA), stone, soil stabilized with a hydraulic binder and geosynthetics are discussed. The conducted research allowed evaluation of the possibility of implementing the DIC method to assess deformation of road materials in laboratory tests. A wide spectrum of studied materials enabled to identify the areas in which the DIC method and the algorithms used in it give a significant advantage over traditional and tensometric measurement methods.

## 2. Fundamentals of DIC

### 2.1. Digital Image Correlation (DIC) System for Registration and Analysis of Displacement/Deformation Fields

Digital Image Correlation (DIC) is a precise, non-contact, and non-interferometric optical method used for measuring the displacement/deformation of a structural element/material subjected to external loading. The idea of the DIC method is based on the principles of continuum mechanics (rigid body mechanics) [12]. The system consists mainly of a digital camera/cameras and specialised computer software (e.g., Istra 4D programme [8]). A camera/cameras is/are used to capture consecutive images (photos/pictures) of the surface of a tested object/material before and during the deformation period. The obtained digital image data (a series of photos) is analysed by the DIC software (computer programs). The mathematical correlation analysis is applied. Finally, a set of displacement/deformation maps for the entire specimen surface is created [4,6,13]. Stress fields can be evaluated from strain fields.

The measurement of displacement/deformation using the DIC system requires the following consecutive stages [8,12]:

- Preparation of the surface of the analysed specimen (patterning)
- Calibration of a device according to the required procedure (using the DIC system calibration)
- Recording images of the examined object/material surface before and during its movement/deformation
- Image analysis (evaluation) using a specialised computer programme (software)
- Visualization of the results.

**Figure 1:**
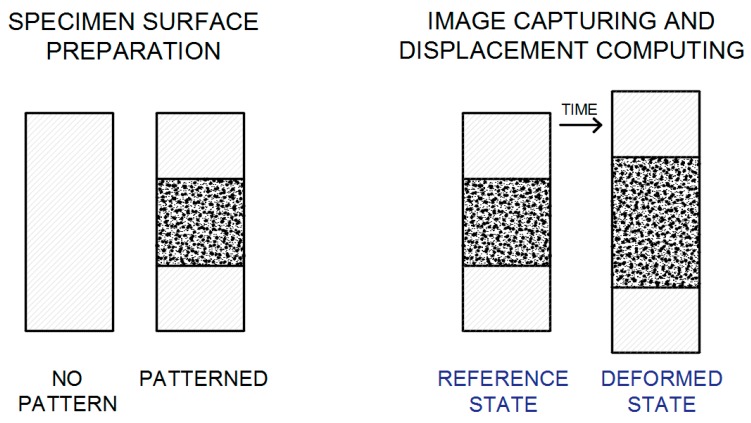
DIC method—Stages [17]. (The Creative Commons Attribution (CC BY 4.0) license (http://creativecommons.org/licenses/by/4.0/)).

**Figure 2:**
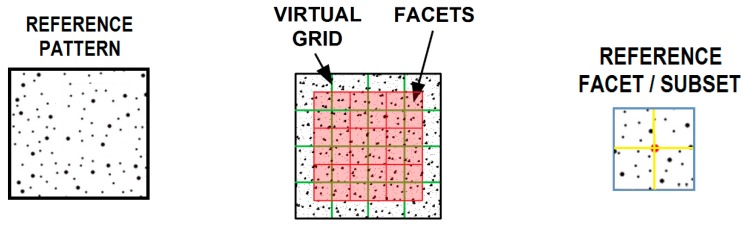
DIC method—Reference image: facets (also called subsets) marked with blue squares and centre points of the facets/subsets located at the intersection of the yellow virtual grid [17,18]. (The Creative Commons Attribution (CC BY 4.0) license (http://creativecommons.org/licenses/by/4.0/)).

**Figure 3:**
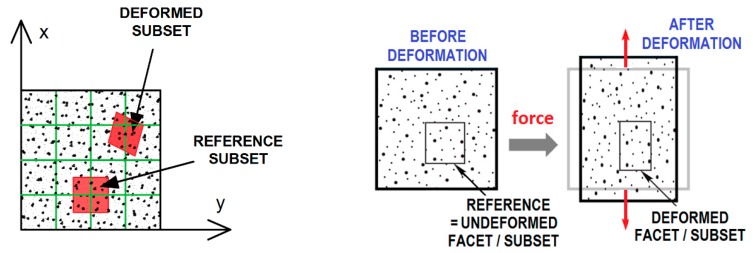
Graphical illustration of the selected region deformation of a scanned surface (a square facet/subset/subimage) in a two-dimensional coordinate system [17,18]. (The Creative Commons Attribution (CC BY 4.0) license (http://creativecommons.org/licenses/by/4.0/)).

At the beginning a tested specimen needs to be prepared by the application of a unique pattern of random speckles/dots to its surface (Figure 1, Figure 2 and Figure 3). The speckle pattern can be the natural texture of the specimen surface or in many cases artificially made by spraying black paint on the white background of the specimen surface (Figure 1, Figure 2 and Figure 3). Other techniques can be applied as well [1]. The speckle-free DIC method was also developed and applied [14]. A special preparation of the specimen surface with the pattern containing the characteristic speckles enables the observation of the changes in the position of these dots during the deformation period [12,15,16,17].

**Figure 4:**
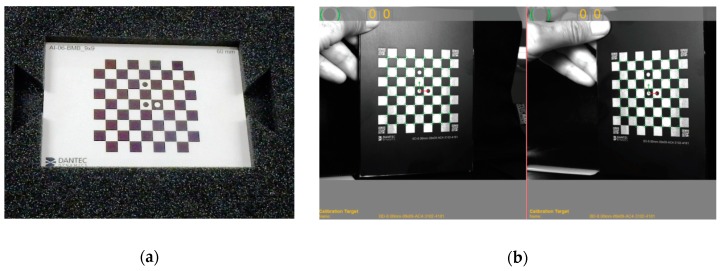
3D-DIC system- Calibration: (**a**) The example of a calibration target; (**b**) Calibration live image [8].

Prior to the loading of the examined object/material surface and before taking photos during its deformation, the calibration/positioning of the device (camera/cameras) according to the appropriate procedure is required, e.g., using a special calibration plate (target) (Figure 4) [8,16].

**Figure 5:**
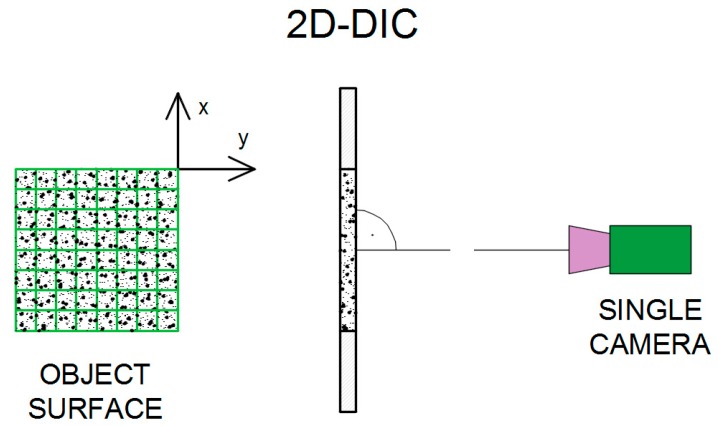
2D-Digital Image Correlation system [17,18,20]. (The Creative Commons Attribution (CC BY 4.0) license (http://creativecommons.org/licenses/by/4.0/)).

**Figure 6:**
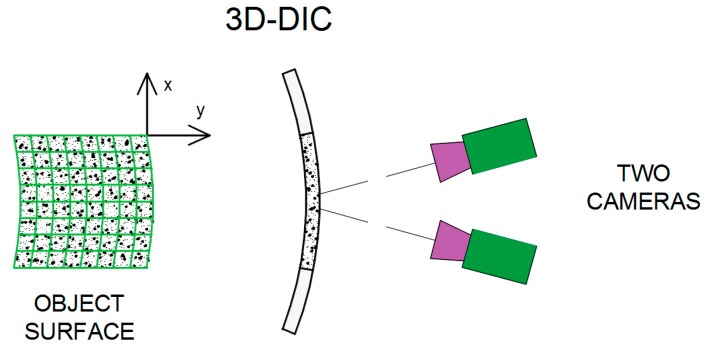
3D-Digital Image Correlation system [17,18,20]. (The Creative Commons Attribution (CC BY 4.0) license (http://creativecommons.org/licenses/by/4.0/)).

Next, a photo/picture/image before loading (“reference image”) and then a series of photos during the deformation period (Figure 1 are taken, applying the proper lighting to the tested specimen. Typically, the DIC system equipped with one or two digital cameras is used (Figure 5 and Figure 6) [8,9,17].

A single-camera system is applied to measure in-plane deformations of the planar objects. A camera is placed with its optical axis normal to the specimen surface (Figure 5). Two-dimensional results of deformation are obtained. Therefore, the system is called two-dimensional digital image correlation (2D-DIC). If the object surface is non-planar, or if 3D deformation occurs after loading, the 2D-DIC method is no longer applicable [1,16,19].

For measuring the displacements/deformations in 3D space at least two cameras are used to record the image of a tested object from different directions [16]. Three-dimensional digital image correlation (3D-DIC) system is presented in Figure 6.

Currently, modern and developed DIC systems, consisting of more than two cameras (three, four, six and even eight cameras) are designed to observe spatial objects from several directions simultaneously. These systems are intended to analyse the material behaviour during vibration, drop tests, crash tests, etc. They are high-speed systems with high sampling frequencies (several thousand fps) contrary to low-speed systems (typical for measuring static deformations) with the sampling frequencies of maximally several fps [4,16].

Nowadays, a more comprehensive analysis is also possible using the DIC systems equipped with a microscope adapter, which allows for microscopic observations of a tested specimen during the deformation period [19].

In the field of experimental mechanics of road materials, the 2D-DIC technique is commonly used as a practical and effective tool for measuring strains [1].

After recording the images obtained from one or more CCD cameras, the subsequent stage of the study is image analysis (evaluation) in a specialised computer programme (e.g., Istra 4D) [4,8]. The DIC system is based on the comparison (correlation) of digital images. A mathematical correlation analysis is applied to compare digital images taken before deformation (“reference image”) and during the deformation process (“deformed image”) (Figure 1, Figure 2 and Figure 3) [16,17,18,21]. At first, a virtual, regular grid of points is placed onto the “reference image”, also called the “specific image” or the “region of interest” (a fragment of the object seen/scanned by the camera before deformation). Each point at the intersection of the virtual grid is the centre of a facet/subset/subimage (“correlation region”/”calculation area”)—a small square region/element of the image (Figure 1 and Figure 2). The minimal facet size is determined by the size of the created pattern in such a way that every facet has to contain white and black colours (e.g., random black speckles/points on the white background) in order to ensure proper correlation. Due to a unique pattern, each facet can be distinguished from other facets and thus can be identified in the “deformed image” (Figure 3). Based on the recorded changes in the location of facets/points in two consecutive images (taken before and during the deformation process), specialised DIC software performs the calculation of the displacement/deformation (for the entire analysed specimen surface) using a correlation algorithm. Depending on the DIC system, NCC (Normalised Cross-Correlation), LSM (Least-Squares Matching), etc., are applied as correlation criteria [1,19].

The last stage of the study is the visualization of the results. The output data is a set of displacement/deformation maps [4,6,8,13,16,18,21].

### 2.2. Advantages and Limitations of Using DIC

The following main advantages of the DIC system can be distinguished [3,8,15,16]:

- The ability to measure the displacements/deformations of samples of various sizes, from micro-samples examined using a microscope to large-size objects such as fragments of bridges
- The possibility of the determination of Poisson’s ratio and Young’s modulus of materials, detection of cracks and damage, the measurement of vibration, modal analysis and the validation of FEM numerical models
- The system is easy to use in laboratory and field conditions.

A comparison of the DIC technique with traditional gauges and sensors techniques (extensometers) is presented in Table 1.

The DIC system also has some limitations, such as:

- The dependence of the system on natural lighting conditions; the need to apply artificial light when registering images with high frequency
- The need to use calibration tables appropriate to the size of the tested sample area and capacious storage media required to archive recorded images and to obtain research results.

## 3. Application of DIC System for Testing Deformation of Road Materials—Results of Own Investigations

In the field of road engineering, the DIC system enables the study of the mechanical parameters of anisotropic and heterogeneous road materials, such as asphalt mixtures (commonly used as a material for pavements), road stones, soils stabilized with a binder, geosynthetics, etc., [5,8,9,22]. The DIC technique is also applied for testing the deformation of structures, e.g., road pavement structures. According to Grygierek et al. [23], this method enables the assessment of the influence of a vehicle wheel load on the resulting deformation of wearing course.

The DIC system is used for various purposes, such as [22,24,25]:

- The evaluation of the correctness of the applied test procedures
- The assessment of local changes in the parameters of materials due to their heterogeneity
- The description of the cracking process
- The validation of theoretical and numerical models.

In the study of road materials conducted at Cracow University of Technology, the 2D digital image correlation system (which provides strain measurements for planar surface specimens) was applied. The following road materials were tested: Hot-mixed asphalt (HMA), soil stabilized with a hydraulic binder, stone (porphyry), and geosynthetics. The results of our own tests of mechanical properties of road materials are presented in this Chapter.

### 3.1. Road Materials Tested

Hot-Mixed Asphalt (HMA) (asphalt concrete) AC11S was used in the study.

The tested soil was silty loam with optimum compaction moisture of 13.8%. As a binder, a two-component road binder was used for stabilizing the soil. The used road binder causes the agglomeration of fines and improves soil characteristics through the waterproofing effect [26]. The test was carried out on 30 cylindrical specimens of soil with the dimensions of 80 mm × 80 mm.

Stone samples were prepared from porphyry rock.

Geosynthetics that perform various functions in civil engineering, e.g., strengthening, filtering, and separating were also investigated [27,28,29,30]. Geosynthetics research was carried out on the three types of reinforcing geogrids: biaxial glass fibre geogrid, biaxial polyester fibre geogrid and triaxial hexagonal polypropylene geogrid. Glass fibre geogrids are designed for the reinforcement of asphalt layers [31,32,33]. Polyester geosynthetics are used in transportation engineering to strengthen a weak soil substrate, reinforce embankments and to stabilise landslides [34,35,36]. Polypropylene geogrids with a hexagonal arrangement of ribs are used for the stabilisation of unbound granular layers by way of interlock with the aggregate [37,38,39].

### 3.2. Preparation of Samples of Road Materials

**Figure 7:**
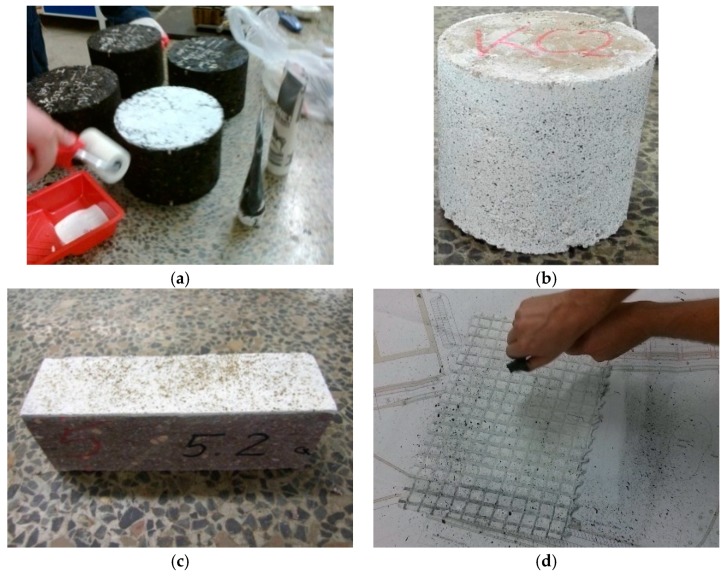
Preparation of the sample surface for testing with the DIC method: (**a**) Painting HMA (Hot-Mixed Asphalt) samples with white paint; (**b**) soil sample prepared to test; (**c**) Stone sample prepared to test—a white background sprinkled with pepper (so called “pepper method”); (**d**) spotting a geosynthetic sample with black colour.

The use of the DIC system requires a proper preparation of road material samples for laboratory tests. It involves painting the selected sample surfaces with white paint and then applying as many black spots as possible, which the DIC system converts into a grid of characteristic elements [38]. The preparation of samples (painting HMA samples with white paint and spotting a geosynthetic sample with black colour) is shown in Figure 7 [24,25]. The prepared samples of soil and stone, covered with a layer of the pattern in the form of a white background with randomly distributed black points, are also presented in Figure 7.

### 3.3. DIC Set-Up

The laboratory stand based on the MTS Landmark servo-hydraulic testing system and the DIC measurement system was used for testing road materials.

The most important elements of the laboratory stand are as follows [24]:

- A servo-hydraulic testing machine station with a thermal chamber
- A digital camera
- Linear lighting
- A controller and a computer for controlling the DIC system
- A place/stand to analyse the results of the DIC system, equipped, for example, with the ISTRA 4D programme.

A servo-hydraulic testing machine can be used to perform static and fatigue tests of road material samples. The accuracy class of force transducers of the testing machine equals 0.5 in a range of the force value from 1% to 100%. The climatic chamber allows testing at temperatures from −40 °C to +60 °C.

**Figure 8:**
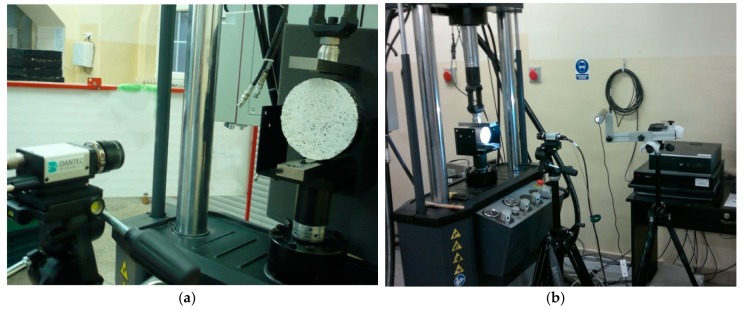
Laboratory stand using the DIC method for the indirect tensile test: (**a**) HMA sample; (**b**) soil sample [40,41].

The laboratory stand prepared for the indirect tensile test is shown in Figure 8.

**Figure 9:**
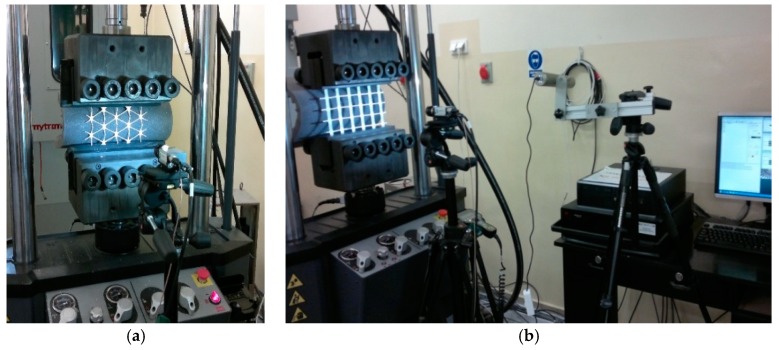
Laboratory stand for geosynthetics testing using the DIC method: (**a**) Sample of hexagonal geogrid; (**b**) sample of orthogonal geogrid [24,38].

Geosynthetics research was carried out on a laboratory stand adapted for this purpose. Special jaws were used to attach geosynthetics samples in a direct tensile test. The laboratory stand during the geosynthetics test is shown in Figure 9.

The DIC set-up used in the study consisted of one camera and an adequate multiple LED light source [4,8]. Photographs were taken during loading of a sample at a determined load increase. The distance of the camera from the observed sample was chosen so that the correct system calibration could be obtained. The post-processing of the measurement results was performed using the ISTRA 4D programme. The determination of the analysed field and the division of this area into the grid of elements were carried out by the software in the first stage of the analysis. As the analysis result, the maps of displacements and deformations of the surface of the tested sample were obtained.

### 3.4. Test Results of Displacements and Deformations of Road Materials Using the DIC Method

Geosynthetics wide-width tensile tests were carried out according to the standard [42]. For other road materials the modulus of elasticity, Poisson’s ratio, compressive strength, and indirect tensile strength were determined according to the standards and procedures [40,41,43,44,45,46,47,48].

#### 3.4.1. Hot-Mixed Asphalt (HMA)

**Figure 10:**
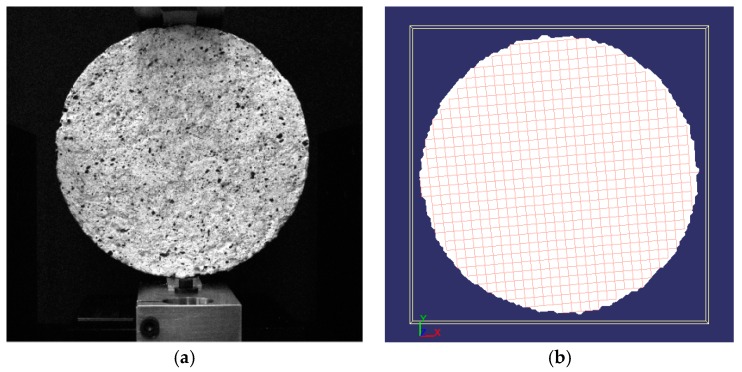
HMA sample: (**a**) View of the sample before testing; (**b**) virtual grid on the surface of the tested sample [25].

The view of the analysed Hot-Mixed Asphalt (asphalt concrete AC11S) sample and the grid of elements on its surface are presented in Figure 10.

**Figure 11:**
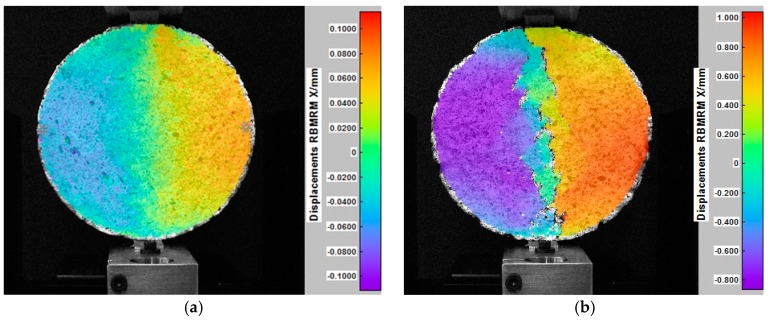
Horizontal displacements of the HMA sample in the indirect tensile test: (**a**) The distribution of horizontal displacements, the load of 0.4 kN; (**b**) final phase of the test—visible vertical cracks of the sample, the load of 3.2 kN [25].

**Figure 12:**
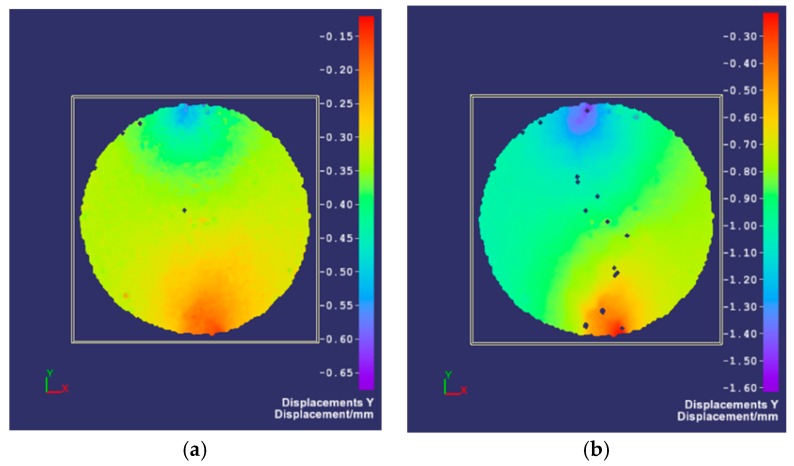
Vertical displacement of the HMA sample in the indirect tensile test: (**a**) The distribution of horizontal displacements, the load of 0.8 kN; (**b**) advanced phase of the test—the formation of local cracks (discontinuities), the load of 1.7 kN [25].

The indirect tensile test was carried out for Hot-Mixed Asphalt (HMA). By registering changes in the position of the grid points on the surface of the sample during the test, its displacements and deformations were determined. Figure 11 shows the horizontal displacements of the analyzed surface of a cylindrical HMA sample in selected phases. Vertical displacement maps generated by the DIC software in the same test are presented in Figure 12.

Using the DIC method, displacements resulting only from sample deformations, excluding displacements associated with the motion of a rigid body, can also be determined for mineral-asphalt mixtures.

Moreover, other important mechanical parameters can be specified, such as Poisson’s ratio for various load levels as well as the deformations accompanying the destruction of the sample. These parameters are used in the mechanistic design of road pavement structures.

In addition, the DIC method can be applied to examine the effect of temperature (its daily and annual changes) on the mechanical parameters of asphalt mixtures during thermo-mechanical tests in a climatic chamber [49,50,51].

#### 3.4.2. Soil Stabilized with a Binder

**Figure 13:**
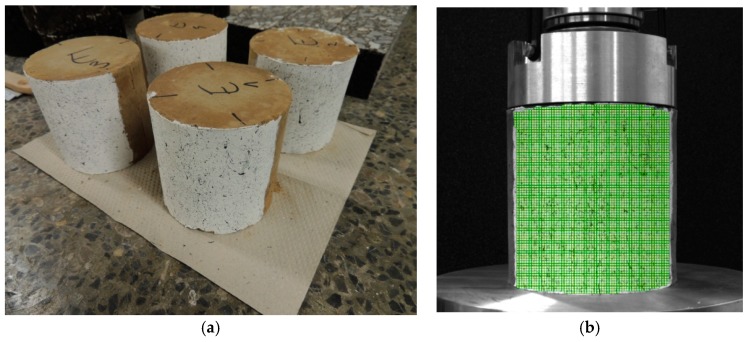
Soil samples: (**a**) View of the samples prepared for the compressive test; (**b**) virtual grid on the surface of the sample [40].

The view of the samples of soil and the virtual grid on the surface of the soil sample are shown in Figure 13.

In the compressive tests, the measured surface was curved. Using the 2D-DIC method, the results should only be considered on the surface perpendicular to the camera (in the axis of symmetry of the sample), where the calibration process were performed. To assess the displacements on the entire curved surface of the sample, it is recommended to use the 3D-DIC system.

The modulus of elasticity under uniaxial compression, modulus of elasticity under indirect tension and Poisson’s ratio were tested for soil stabilized with a binder. The results of the mechanical properties of chemically stabilized soil are summarized in Table 2.

The mean value of Young’s modulus of the tested soil was equal to 670 MPa in a compressive test and 583 MPa in an indirect tensile test. The value of Poisson’s ratio was equal to 0.22 with the coefficient of variation 11%. According to the results, the indirect tensile strength is equal to about 10% of the value of compressive strength. This result is consistent with the results presented in the literature [40,41].

**Figure 14:**
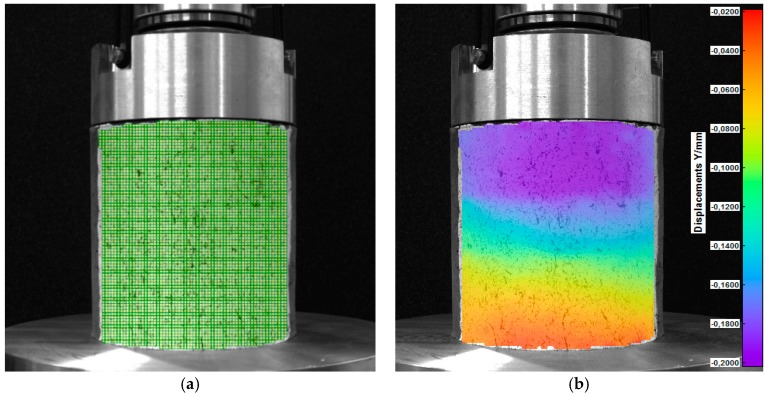
Soil sample under uniaxial compression: (**a**) Virtual grid of the DIC method, (**b**) vertical displacements of the sample in the compressive test [40].

**Figure 15:**
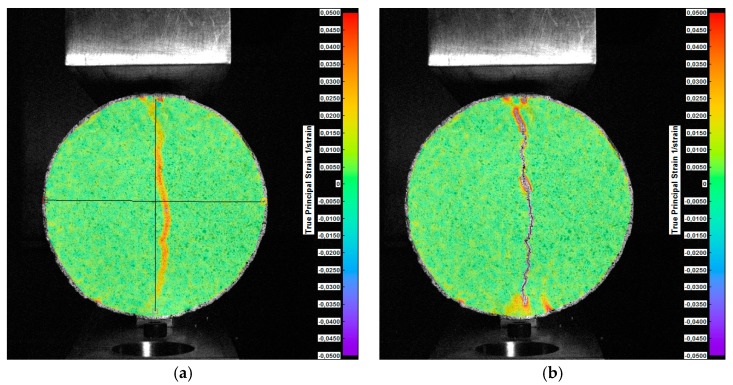
Maps of maximal true principal strains of the soil sample surface in the indirect tensile test: (**a**) Phase immediately before cracking; (**b**) sample with cracks [41].

The maps of displacements and principal strains of the specimen surface obtained in the compressive test and in the indirect tensile test are shown in Figure 14 and Figure 15, respectively.

#### 3.4.3. Stone (porphyry)

**Figure 16:**
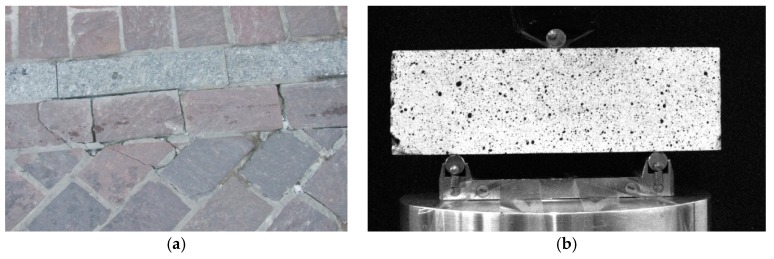
Stone (porphyry) sample: (**a**) Stone slab in the road pavement cracking due to bending; (**b**) stone beam prepared for a three-point bending test using DIC [25].

**Figure 17:**
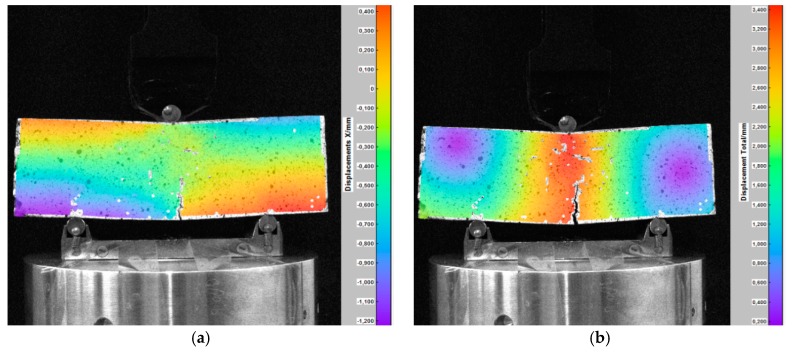
Horizontal displacements of a rock sample in a three-point bending test: (**a**) Initial phase of the test; (**b**) total displacement; final phase of the test - a clearly visible vertical crack in the sample.

Stone (porphyry) beams taken from the stone slab pavement (see Figure 16) were examined in a three-point bending test using the DIC technique. The test results in the form of displacement maps are shown in Figure 17.

The mechanical parameters obtained in the tests are related to the durability of a road pavement made of stone slabs. In many cases, the insufficient tensile strength in bending of stone slabs causes a premature failure of a road pavement (see Figure 16). Therefore, the obtained parameters can be used in both theoretical and numerical analyses as well as during the design of pavement structures [40,41].

#### 3.4.4. Biaxial Glass Fibre Geogrid and Polyester Geogrid

**Figure 18:**
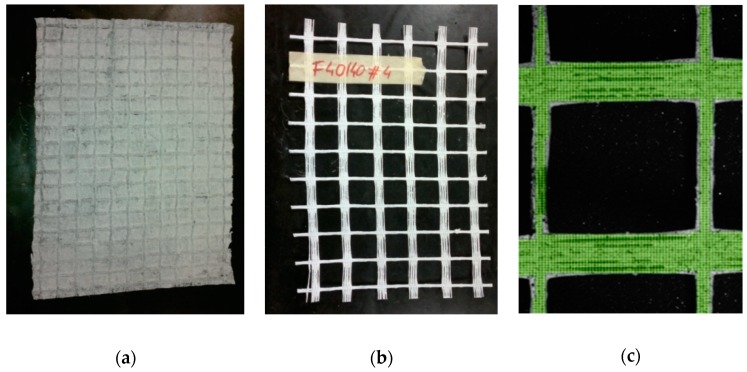
Geogrids samples prepared for testing: (**a**) Glass fibre geogrid; (**b**) polyester geogrid; (**c**) virtual grid for the polyester geogrid (geosynthetics) [52].

The view of the analyzed samples of biaxial orthogonal geogrids and the grid of elements on the polyester geogrid surface are shown in Figure 18.

In the tests of the samples of geogrids, the values and distributions of linear strains were obtained. The tests were carried out according to the standard [42]. Table 3 and Table 4 present a comparison of the values of vertical linear strains determined using two measurement techniques for various load levels. The obtained results show no significant differences, indicating the effectiveness of the DIC system in this study.

**Figure 19:**
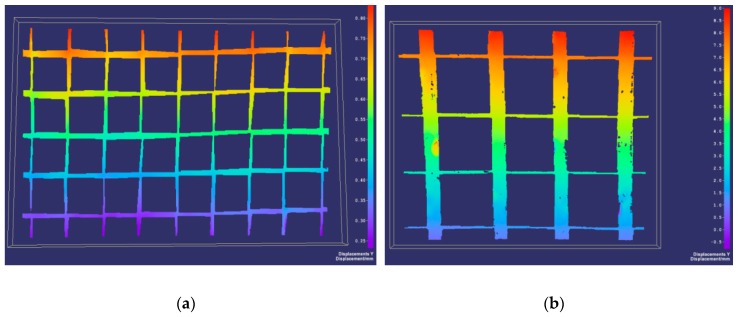
Vertical strains of biaxial geogrids: (**a**) Maps of the distribution of vertical strains on the surface of the glass fibre geogrid sample; (**b**) maps of the distribution of vertical strains on the surface of the polyester geogrid sample [52].

The selected results of laboratory tests in the form of colored maps of strains are presented in Figure 19.

The DIC method enables to determine the field of deformations for the entire surface of the geogrid sample along with the solution of the issue of principal strains and principal directions. This is a great advantage of this method because it is unattainable using traditional measuring methods—extensometers (strain gauges). In addition, the DIC method allows for the visualization of deformations, which are specific for geogrids as heterogenic and anisotropic structures.

#### 3.4.5. Triaxial Polypropylene Geogrid

**Figure 20:**
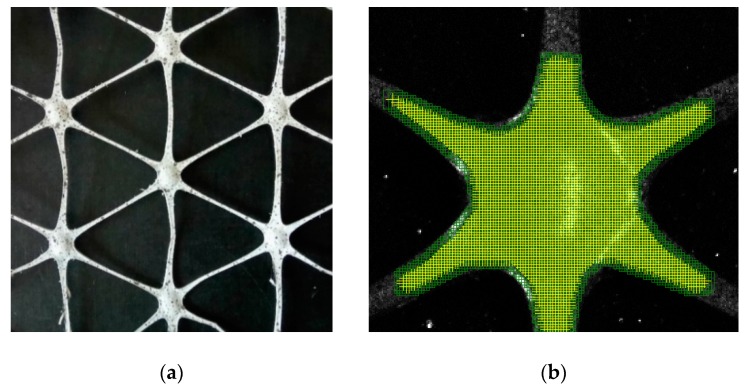

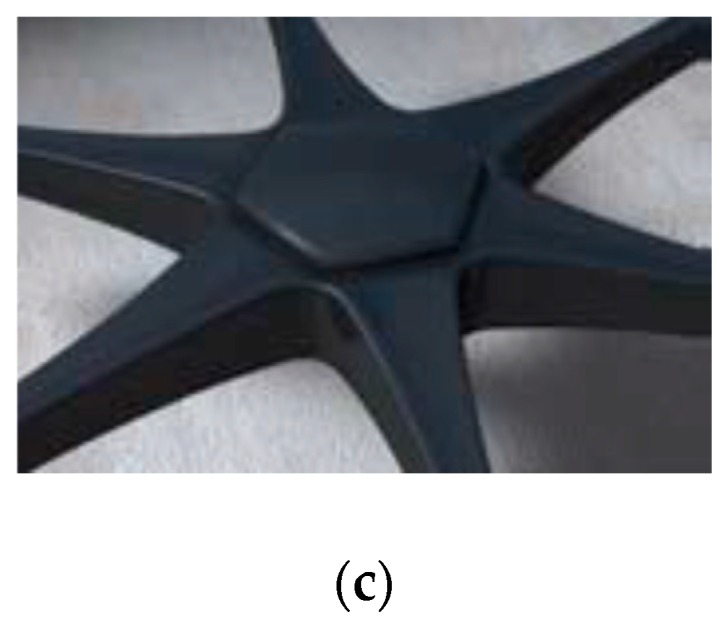
Triaxial hexagonal polypropylene geogrid: (**a**) Geogrid sample prepared for testing (after painting with white paint and spotting with black colour); (**b**) virtual grid of a hexagonal geogrid node; (**c**) geogrid node before painting [38].

The view of the triaxial hexagonal polypropylene geogrid sample and the grid of elements on its surface are shown in Figure 20.

**Figure 21:**
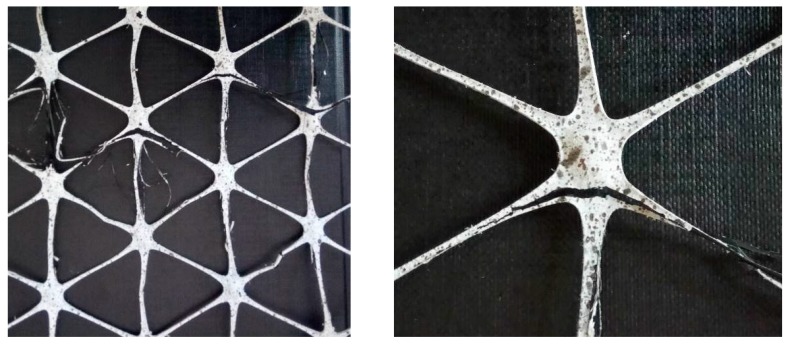
The central part of the polypropylene geogrid sample after the tensile test with a visible destroyed geogrid node [38].

Laboratory tests allowed to determine load-strain curves as well as selected mechanical parameters of the tested samples. During the tensile test, when the material was loaded, it was observed that all the samples were destroyed by breaking the nodes. The central part of the sample after the test is shown in Figure 21.

**Figure 22:**
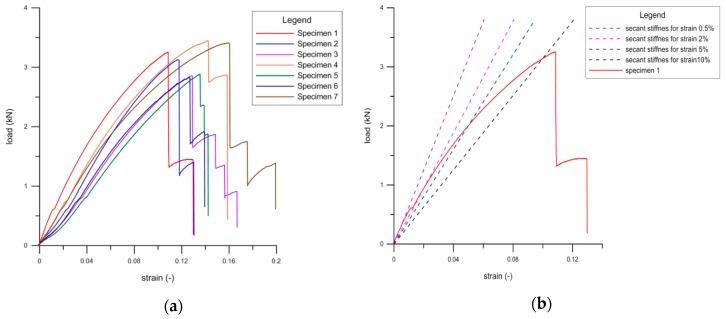
Polypropylene geogrid samples: (**a**) Load-strain curves; (**b**) secant stiffness lines [38].

Load-strain curves obtained in the tension test for geogrid samples are shown in Figure 22. Secant stiffness of a polypropylene geogrid was also determined (according to the standard [42]). A graph showing secant stiffness lines for the assumed levels of strains 0.5%, 2%, 5%, 10% is presented in Figure 22. The results of the tensile strength and secant stiffness are summarized in Table 5.

**Figure 23:**
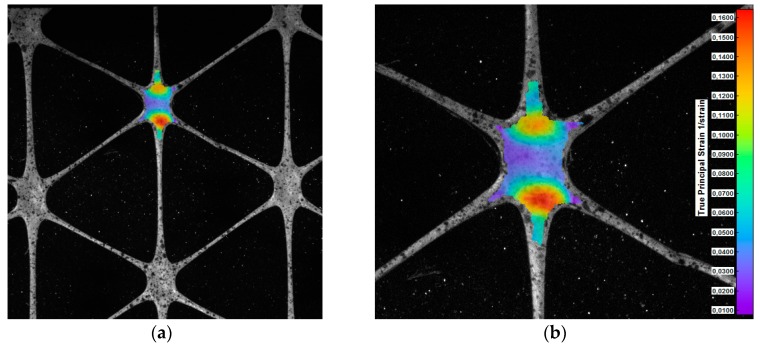
Map of the distribution of the principal strains 1 on the surface of the polypropylene sample: (**a**) Geogrid view; (**b**) close-up of the node [38].

Due to the observed damage character of the geogrids, special attention was paid to the behaviour of the nodes in the tensile test. The displacement measurements and the analysis of the deformation distribution were carried out using the DIC method. A map showing the distribution of principal strains on the surface of a polypropylene sample is presented in Figure 23.

The obtained strain maps point out that the largest of the principal strains appear in zones on the edges of the nodes of the tested polypropylene geogrids. Therefore, maximum equivalent strains and stresses can occur in these areas, which explains the observed nature of the grid damage in the form of cracks on the edges of the nodes. Cracks are perpendicular to the direction of the maximum principal strains. The destruction of the sample occurred with the maximum principal strain 20% greater than the tensile/vertical strain measured in the base of the extensometer [38].

## 4. Discussion

In recent years, researchers have been working on extending the range of application of the DIC (Digital Image Correlation) method. Most of the efforts are focused on the development of multi-camera DIC systems, in which data obtained from individual systems are stitched together in a common coordinate system [7].

With a history of more than 30 years, DIC has been developed into an effective and flexible optical technique to measure surface deformations at macro-, micro-, and even nanoscale. Numerous applications in broad fields have already proven that it is a practical and indispensable tool for measuring deformations [1].

Optoelectronic, non-contact systems are increasingly being used to monitor materials and structures in civil engineering. This also applies to materials used in road construction. The study of mechanical parameters of road materials is an important issue from the point of view of the condition and durability of road pavements. The implementation of the DIC method in the laboratory tests of road materials confirms the possibilities of wide applications of this method.

## 5. Conclusions

The results of the conducted research allow the formulation of the following conclusions:

- The Digital Image Correlation (DIC) method implemented on a properly prepared laboratory setup enables the effective measurements of deformation of road materials under various load conditions.
- The great advantage of the DIC technique is the ability to determine displacements and deformations in any direction and at any point (for the entire surface of the sample) along with the solution of the issue of principal strains values and principal directions. This is unattainable using traditional measurement methods (strain gauges).
- The DIC method can visualize deformations of road materials, which are specific/complicated in shape (e.g., heterogenic and anisotropic geogrids).
- The numerical algorithm used in the DIC system eliminates the displacements associated with the motion (movement) of a rigid body (sample) during the test. This reduces the problem of slipping out a geosynthetic sample from the jaws of a testing machine.
- The DIC method gives the possibility to validate the results obtained on the basis of numerical models [24].
- The constantly improved DIC method opens new research fields for non-contact determination of deformations of modern, more complex road materials under various load conditions.

## Figures and Tables

**Table 1:** Comparison of the DIC method with measurements by using extensometers [5,8,16].

DIC System	Extensometers
Non-contact measurement	Contact measurement
Unlimited number of deformation measurements	An extensometer can be used only once (a glued extensometer cannot be peeled off without damaging it)
The possibility of testing samples of any shape and material; the tested surface of the sample does not have to be flat	A surface on which the extensometer is glued has to be flat
The ability to measure deformation in all directions (along X-axis, Y-axis, Z-axis), on a plane or in three-dimensional space	The ability to measure deformation only in the chosen direction
Full-field deformation analysis	Results of the deformation at selected points of the sample, i.e., at the points where the sensors are attached
A measurement of the real maximum displacements and deformations	A measurement limited by the maximum value of the deformation of an extensometer
A quick preparation of a random pattern of black dots on the white background on the sample surface by spraying paint	A time-consuming process of placing the extensometer on the surface of a sample (gluing, etc.)
The need to clean the surface of a sample before testing	

**Table 2:** Mechanical properties of the tested soil [40].

Material Properties	Mean Value	Coefficient of Variation
Modulus of elasticity under uniaxial compression E_c_ (MPa)	670	22%
Modulus of elasticity under indirect tension E_it_ (MPa)	583	34%
Poisson’s ratio (-)	0.22	11%
Compressive strength R_c_ (MPa)	2.64	5%
Indirect tensile strength R_it_ (MPa)	0.27	24%

**Table 3:** Linear vertical strains (cross machine direction)—glass fibre geogrid; average values of strains from 5 samples [52].

Load	Tensometric Technique	DIC (Averaging for a 60 mm Base)	Ratio of Results
18.49 kN	200 × 10^−4^	209 × 10^−4^	1.05
22.10 kN	250 × 10^−4^	273 × 10^−4^	1.09
16.89 kN	300 × 10^−4^	324 × 10^−4^	1.08

**Table 4:** Linear vertical strains (machine direction)—polyester geogrid; average values of strains from 5 samples [52].

Load	Tensometric Technique	DIC (Averaging for a 60 mm Base)	Ratio of Results
2.64 kN	300 × 10^−4^	336 × 10^−4^	1.12
6.27 kN	600 × 10^−4^	654 × 10^−4^	1.09
10.83 kN	900 × 10^−4^	965 × 10^−4^	1.07

**Table 5:** Mechanical properties of the tested polypropylene geogrid [38].

Material Properties	Mean Value	Standard Deviation	Coefficient of Variation
Tensile strength (kN/m)	16	1.2	8%
Tensile strain at maximum tensile load (-)	13%	1.5%	12%
Secant stiffness at 0.5% strain (kN/m)	167	64.7	39%
Secant stiffness at 2% strain (kN/m)	142	48.5	34%
Secant stiffness at 5% strain (kN/m)	150	28.4	19%
Secant stiffness at 10% strain (kN/m)	134	14.1	11%
